# Systems pharmacology to investigate the interaction of berberine and other drugs in treating polycystic ovary syndrome

**DOI:** 10.1038/srep28089

**Published:** 2016-06-16

**Authors:** Yu Wang, Xin Fu, Jing Xu, Qiuhong Wang, Haixue Kuang

**Affiliations:** 1 Key Laboratory of Chinese Materia Medica (Ministry of Education), Heilongjiang University of Chinese Medicine, 150040, Harbin, P.R. China

## Abstract

Polycystic ovary syndrome (PCOS) is a common multifactorial endocrine disorder among women of childbearing age. PCOS has various and heterogeneous clinical features apart from its indefinite pathogenesis and mechanism. Clinical drugs for PCOS are multifarious because it only treats separate symptoms. Berberine is an isoquinoline plant alkaloid with numerous biological activities, and it was testified to improve some diseases related to PCOS in animal models and in humans. Systems pharmacology was utilized to predict the potential targets of berberine related to PCOS and the potential drug-drug interaction base on the disease network. In conclusion, berberine is a promising polypharmacological drug for treating PCOS, and for enhancing the efficacy of clinical drugs.

Polycystic ovary syndrome (PCOS) is an endocrine disorder characterized by anovulation, hyperandrogenism and polycystic ovaries in ultrasonic scanning, which affects proximate 5–15% in women of childbirth age^1^,^2^,^3^,^4^,^5^,^6^.

The clinical manifestations of PCOS are heterogeneous and diversity in androgen excess (hyperandrogenemia, hirsutism, acne and alopecia), irregular menstrual cycle, infertility, insulin resistance and obesity. PCOS patients also have a high risk for the development of severe metabolic disorders, such as cardiovascular disease, type 2 diabetes, metabolic syndrome and endometrial cancer^7^.

The pathogenesis and mechanism of PCOS remain unclear. PCOS is currently considered as a mainly hyperandrogenic disorder^8^. Many PCOS patients are overweight or obese, and a mainly abdominal fat distribution is particularly common in these women^9^,^10^. Insulin resistance has been implicated in the pathogenesis of anovulation and infertility in PCOS, and abnormalities in insulin action have been observed in a variety of reproductive tissues in PCOS women^11^, 50–70% of patients with PCOS show insulin resistance and compensatory hyperinsulinism^12^. Escobar proposed that a vicious circle has existed in PCOS women, that may start during their early stages of life or even in babyhood, whereby androgen excess favoring the abdominal deposition of fat further facilitates androgen secretion by the ovaries and adrenals in PCOS patients^13^.

Given its heterogeneous clinical manifestations, the treatment of PCOS is not unified, but instead depends on each phenotypic features and reproductive desire. Weight reduction and lifestyle modifications are highly recommended therapies that improve the metabolic status, ovulation regularity and protection of PCOS patients from cardiovascular diseases^14^, unless other issues indicate that drug or hormonal interventions are superior. So the drugs used in clinical treating for PCOS are numerous including combined oral contraceptives (COCs), antiandrogenic progestins, insulin-sensitizing drugs, and so forth. Generally, these drugs only treat the respective single phenotypic feature^7^.

Berberine is an isoquinoline plant alkaloid isolated from several plants such as *Hydrastis canadensis*, *Berberis vulgaris*, *Coptis chinensis* and *Berberis aristata*. Berberine has multiple pharmacological properties, including anti-microbial, glucose-lowering and cholesterol-lowering, anti-tumoral and immune- modulating activities^15^. Several studies show that berberine can increase glucose uptake and reduce insulin resistance *in vitro*^16^,^17^. An increasing number of clinical studies also emphasize the positive role of berberine in treating PCOS^18^,^19^,^20^,^21^,^22^. Berberine is a high-affinity substrate for P-glycoprotein (P-gp), organic cation transporters and some cytoch-rome P450 isoenzymes, which all have crucial roles in the transshipment and metabolism of drugs, so berberine was reported to increase the blood concentration of other drugs transferred by these pathways to enhance drug effect^23^,^24^,^25^. Some clinical studies also began to evaluate the cooperation of berberine and other drug^26^. To date, no serious adverse effects have been reported for BBR, apart from gastrointestinal side effects^27^.

Systems pharmacology is defined as an approach to translational medicine that combines computational and experimental methods to elucidate, validate and apply new pharmacological concepts to the development and use of drugs^28^. The previous computational methods for the identification of drug-target interactions depended on some single study such as target fishing, compound profiling, ligand similarity search^29^,^30^ and “omics” approach. In contrast to previous methods, systems pharmacology provides an integrated “systems-level” approach to determi-ning mechanisms of action of drugs in animal models and in patients^28^. Systems pharmacology is widely used in drug discovery, target prediction and mechanism research, especially in traditional herbal medicine^31^,^32^. This article applies systems pharmacology to investigate the mechanism of berberine and its interaction with other clinical drugs in treating PCOS at the target level.

## Results and Discussion

### Candidate targets of PCOS screening and pathway-target network analysis

The targets were selected using our research strategy and verified in the UniProt database (http://www.uniprot.org/). A total of 130 candidate targets (Table S1) of PCOS were validated finally. After inputting these targets to Database for Annotation, Visualization and Integrated Discovery (DAVID) database, 84 targets were enriched to 13 Kyoto Encyclopedia of Genes and Genomes (KEGG) biological pathways and results were shown in Table S2. Six biological pathways including neuroactive ligand-receptor interaction (NLI), adipocytokine signaling pathway (ASP), insulin signaling pathway (ISP), PPAR signaling pathway (PSP), type II diabetes mellitus (TDM) and steroid hormone biosynthesis (SHB) show extreme significant P value (P < 0.002). NLI demonstrates various interactions between the ligands and receptors, and the endometrial gene expression altering among PCOS patients is related to NLI as reported in clinical study^33^. ASP and PSP are correlated with leptin and adiponectin production, which regulates fatty acid oxidation and glucose uptake. ISP and TDM are related to insulin resistance; SHB is involved in biosynthesis of androgens, estrogens, progesterones and glucocorticoids. The six pathways cover all three main potential mechanisms including sex hormones disorder, insulin resistance and abnormal lipid metabolism.

**Figure 1:**
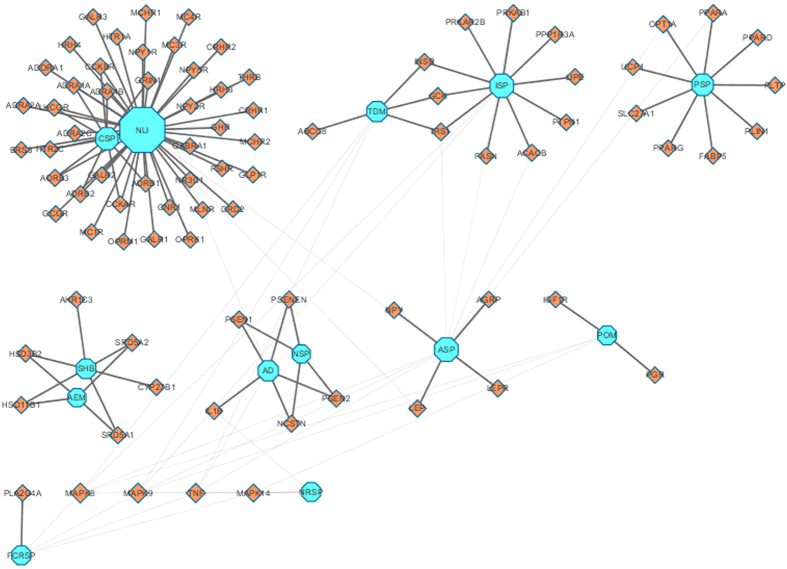
Target-pathway network. The target-pathway network was constructed by linking the potential targets and their biological pathways. The nodes represent targets (orange diamonds) and pathways (blue octagon).

To decipher the potential mechanism and potential significant targets of PCOS, a pathway-target network was constructed based on potential targets and their acting pathways. As shown in Fig. 1, the pathway-target network contains 97 nodes (13 pathways and 84 potential targets) and 130 edges. The mean degree value (the number of the target associated with it) of biological pathways was 10. The degree of NLI, ASP, ISP and PSP are 44, 12, 12 and 9; the betweeness centrality of NLI, SHB and ASP is 0.71, 0.67 and 0.51, that are the top-ranks in pathway-target network respectively. It described the importance of these pathways. Additionally, the results indicate that some targets have been hit by multiple pathways in the pathway-target network. Mitogen-activated protein kinase 8 (MAPK8), mitogen-activated protein kinase 9 (MAPK9), tumor necrosis factor (TNF) and mitogen-activated protein kinase 14 (MAPK14) are linked by 6, 6, 5 and 3 pathways. Given their important positions in the pathway-target network, the MAPK family (MAPK8, MAPK9 and MAPK14) may be the key targets in PCOS mechanism. The MAPKs are mediators of signal transduction from the cytosol to the nucleus^34^, which is involved in androgen biosynthesis and insulin resistance in PCOS^35^,^36^.

**Figure 2:**
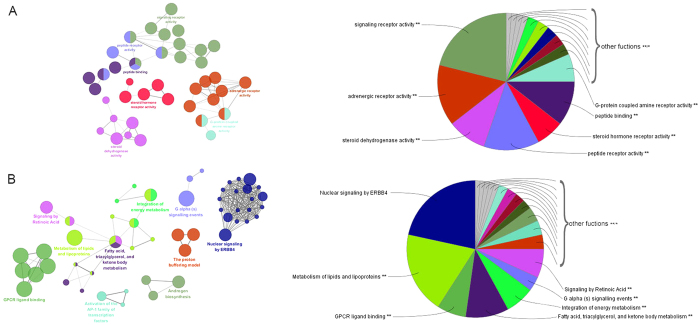
ClueGO analysis of the candidate targets. Functionally grouped network of enriched categories was generated for the target genes. GO terms are represented as nodes, and the node size represents the term enrichment significance. The node pie charts represent the molecular function and reactome analysis of these targets. Only the most significant term in the group was labeled. (**A**) Representative molecular function interactions among candidate targets. (**B**) Representative reactome analysis interactions among candidate targets.

As a widely used Cytoscape plugin, ClueGO^37^ was used to further identify the biological functions of these 130 potential targets in biological networks. As shown in Fig. 2, the results were divided into two strata: molecular functions and the reactome analysis. Specially, the molecular functions were mainly consisted of five groups: signaling receptor activity, adrenergic receptor activity, steroid dehydrogenase activity, peptide receptor activity, steroid hormone receptor activity, peptide binding, G-protein coupled amine receptor activity and monocarboxylic acid binding, which indicated that most potential targets were related to signaling receptor activity, peptide receptor activity and peptide binding (Fig. 2A). The reactome of the targets were mainly related to activation of nuclear signaling ERBB4, metabolism of lipids and lipoproteins, GPCR ligand binding, fatty acid, triacyglycerol, and ketone body metabolism, intergration of energy metabolism and signaling by retinoic acid (Fig. 2B). Finally, we found that most of the targets were related to the activation of GPCR ligand binding, metabolism of lipids and lipoproteins and fatty acid, triacyglycerol, and ketone body metabolism. These biological functions have been linked to material transmembrane transport, lipids metabolism and hormone metabolism.

### Finding known drug targets and potential protein targets of berberine

In the similar manner, we identified 30 clinical drugs for treating PCOS 30 drugs for treating PCOS from the network database and literatures. These drugs include four combined oral contraceptives (COCs), seven antiandrogenic progestins, four antiandrogens, five insulin-sensitizing drugs, three stains, two aromatase inhibitors and five other drugs (Table 1). COCs demonstrate hormonal action at central and peripheral levels simultaneously: suppressing the luteinizing hormone release and subsequently decrease ovarian androgen production; increasing the liver’s production of sex hormone binding protein, which decreases the free androgens in plasma^38^. The reduced androgen synthesis and the peripheral block of androgen receptors represents additional activities acting on hyperandrogenism^39^. The efficacy of antiandrogenic progestins is due to antagonize androgen receptor or inhibit 5a -reductase activity to reduce androgen level^38^. Antiandrogens are the most effective drugs currently available for androgen excess by blocking androgen receptor blockers or inhibiting 5 a-reductase given the mechanism of action^40^. Insulin-sensitizing drugs act by improving insulin sensitivity to compete with insulin resistance and subsequently prevent cardiovascular disease and type 2 diabetes. Statins benefit in PCOS patients by improving chronic inflammation and lipids metabolism, androgen excess, oxidative stress and metabolic parameters^41^. Aromatase inhibitors block the conversion of testosterone and androstenedione to estradiol and estrone, respectively. The decrease of estrogens activity releases the hypothalamus via negative feedback, allowing for the release of follicle-stimulating hormone and luteinizing hormone^42^. From the results of DrugBank database, 25 drugs obtained 17 targets related to PCOS. Therefore, most of the currently available clinical drugs for treating PCOS can only affect limited number of targets current clinical.

Fifteen PCOS-related potential protein receptors of berberine were identified by contrasting and merging the results from PharmMapper and the TCMSP database with the PCOS candidate targets. The targets were shown in Table 2 with PharmMapper Fit score respectively. These targets can be divided into three categories based on their functions. Androgen receptor (AR), estrogen receptor (ESR1) and progesterone receptor (PGR) are nuclear hormone receptors that are directly involved in biosynthesis and conversion of androgens. Retinoic acid receptors (retinoic acid receptor RXR-alpha (RARA) and retinoic acid receptor gamma (RARG)) are also related to androgen biosynthesis^43^. Aldo-keto reductase family 1 member C3 (AKR1C3) can interconvert active androgens, estrogens and progestins with their cognate inactive metabolites. Insulin receptor (INSR) and dipeptidyl peptidase 4 (DPP4) are involved in insulin resistance, in which DPP4 is a novel emerging target against hyperglycemia^44^. Glucocorticoid receptor (NR3C1), corticosteroid 11-beta-dehydrogenase isozyme 1 (HSD11B1) and methionine aminopeptidase 2 (METAP2) are linked to lipid metabolism^45^,^46^. Tyrosine-protein phosphatase non-receptor type 1 (PTPN1), thyroid hormone receptor beta (THRB) and beta-2 adrenergic receptor (ADRB2) are simultaneously implicated with insulin resistance and obesity^47^,^48^,^49^, and MAPK14 acts on androgen biosynthesis and insulin resistance as previously mentioned.

### Molecular docking

**Figure 3:**
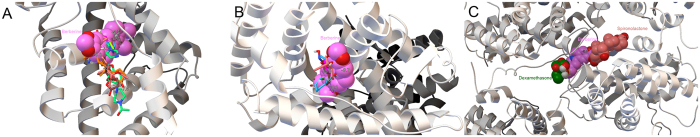
Drugs dock in the same site in targets. (**A**) Docking site between AR and drugs. (**B**) Docking site between PGR and drugs. (**C**) Docking site between NR3C1 and drugs.

**Figure 4:**
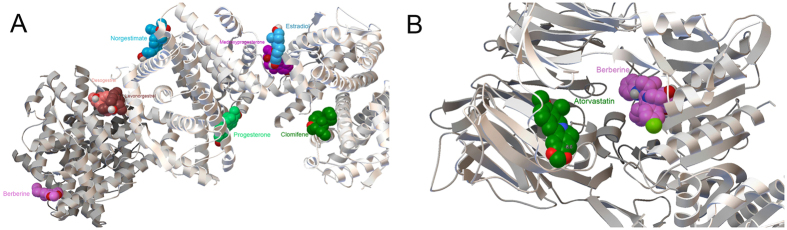
Drugs dock in the different sites in targets. (**A**) Docking site between ESR1 and drugs. (**B**) Docking site between DPP4 and drugs.

To confirm the valid bonding effects between berberine and its predicted targets, molecular docking was evaluated using the berberine-target binding energy with the clinical drug-target binding energy acting as contrast. Additionally some studies suggest that AMP-activated protein kinase (AMPK) plays a central role in pathways which berberine modulates cellular processes^50^, so we also selected three subunit proteins from AMPK family including AMPKA1, AMPKA2 and AMPKB1 (PDB ID: 4RED, 2H6D and 4CFE) to measure the combination ability with berberine using AutoDock Vina. AMPK is a cellular energy sensor that, upon activation, stimulates catabolic processes (such as fatty acid oxidation, glucose uptake, lipolysis) while inhibits anabolic processes (such as gluconeogenesis, fatty acid synthesis, cholesterol synthesis)^50^. As shown in Table 3, berberine shows medium combining capacity for docking all 18 targets and has an analogous binding energy with clinical drugs with the same targets. Therefore, berberine can efficiently act on the 18 targets, and it shows the identical combining capacities comparing with the clinical drugs. To investigate drug interactions in the same target, the docking results of AutoDock Vina were opened in AutoDockTools (ADT) with the conformation of minimum binding energy, which is the most stable conformation. The most stable conformations of all drugs targeting on AR, PGR and NR3C1 bonded target in one identical active pocket (Fig. 3). As a result of the existence of competitive binding on the same active site, we speculate that these drugs trend to show competitive effects in these targets which probably could weaken target toleration to enhance drug effect in a long-time therapy and alleviate adverse drug reactions. By contrast the most stable conformations of those drugs that targeted the ESR1 and DPP4 bonded targets were all drugs acting on ESR1 and DPP4 were found in different active pockets (Fig. 4), so these drugs show a synergic effect in these targets.

### Drug-target network

**Figure 5:**
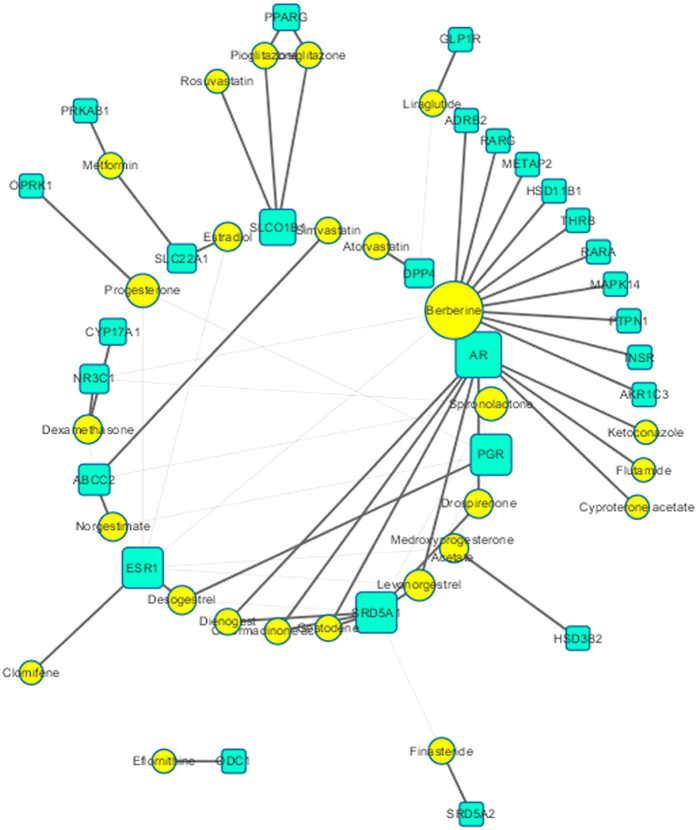
Target-pahtway network. The drug-target network was constructed by linking the drugs and targets. The nodes represent durgs (yellow ellipses) and targets (green round rectangles).

To determine the target distribution of drugs, drug-target network was constructed based on all drugs including berberine and their acting targets. As shown in Fig. 5, the drug-target network contains 53 nodes (31 drugs and 22 targets) and 73 edges. Berberine has the highest degree and betweeness centrality of 15 and 0.5, respectively, thereby indicating that berberine has the most important position in the network. The degrees of AR, 3-oxo-5-alpha-steroid 4-dehydrogenase 1 (SRD5A1), ESR1 and PGR are 10, 8, 8 and 8 respectively, which indicate that shows those drugs affecting hormone receptors are commonly used in clinical applications.

After combining the molecular docking results, we conjectured that berberine probably benefit PCOS patients around the center mechanism by activating AMPKs and MAPKs, and play its role for PCOS with all three phenotypes: activating ESR1 and PGR, suppressing AR, AKR1C3 and retinoic acid receptors to reduce androgen level of ovarian and plasma; activating INSR, THRB and PTPN1, inhibiting DPP4 to compete insulin resistance; activating NR3C1, THRB, PTPN1, inhibiting HSD11B1 and METAP2 to reduce body weight. Comparing with clinical drugs, berberine is likely to act on multiple targets and phenotypes in treating PCOS. As using combination drug therapy of berberine and other drugs, berberine could help clinical drugs access better efficacy by expressing synergy on the same target and acting on other targets of the disease network. In the meantime berberine seemingly plays a role of reducing the side effects by the competition on the same target, such as competitively antagonizing AR to lower teratogenic effect of antiandrogens.

## Conclusion

PCOS is a chaotic disease with multiple factors and various clinical manifestations, so we probably need multi-target drug or multi-drug combination against PCOS. The modulation of a single drug target can be therapeutically insufficient, particularly in complex neuropsychiatric conditions, infectious diseases and cancers. Instead, it is frequently necessary for a drug to simultaneously engage two or more targets for therapeutic efficacy^51^. Despite its low bioavailability, berberine is a promising polypharmacological drug for PCOS as established by potential targets distribution and several studies in humans. The bioavailability of berberine may be increased through co-administration with absorption enhancers and development of berberine analogues or derivatives. Once berberine is administered concurrently with other drugs, clinical dose adjustment based on drug monitoring is recommended because of potential interactions between drugs.

## Materials and Methods

Our protocol processed with four main strategies: (1) finding candidate targets related to PCOS and known targets of clinical drugs; (2) finding potential candidate targets of berberine related to PCOS; (3) constructing pathway-target network and drug-targets network, then analyzing these networks; (4) conducting molecular docking and analyzing docking results.

### Finding candidate targets of PCOS and known drug targets

The mechanism of PCOS is undefined, so we searched potential candidate targets with searching keywords of hyperandrogenemia, hirsutism, acne, alopecia, menstrual disease, infertility, insulin resistance and obesity which are main phenotypic features of PCOS from TTD (http://bidd.nus.edu.sg/group/ttd/)^52^, PharmGkb (www.pharmgkb.org)^53^ and OMIM (http://www.omim.org/)^54^ database. The clinical drugs were collected by Endocrine Society’s practice guidelines for the diagnosis and treatment of PCOS^55^ and literatures searching. Then the targets of these drugs were searched from DrugBank (http://www.drugbank.ca/)^56^.

### Finding potential targets of berberine

**Figure 6:**
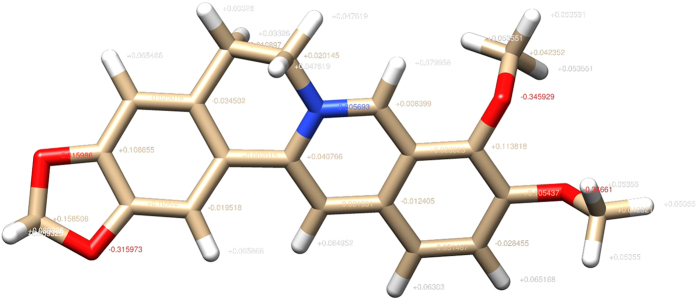
The structure of berberine molecule with added Gasteiger partial charges using Chimera 1.10.2.

The spatial structure of berberine was downloaded from Pubchem compound database^57^(Compound ID: 2353) with SDF format. The berberine structure then was optimized by assigning Gasteiger partial charges with AMBER ff14SB force field using Chimera 1.10.2 (Fig. 6). The potential targets of berberine were predicted by PharmMapper and TCMSP Database respectively.

PharmMapper (http://lilab.ecust.edu.cn/pharmmapper/index.php)^58^ is a web-based tool designed to predict potential drug target candidates for any given small molecule via a ‘reverse’ pharmacophore mapping approach. The model is supported by a large repertoire of pharmacophore database composed of more than 7,000 receptor-based pharmacophore models extracted from Target-Bank, DrugBank, BindingDB and PDTD. It predicts the best mapping poses for a given query molecule against all the pharmacophore models using ligand-protein reverse docking approach. The result lists the topN best-fitted hits with their respective aligned poses and target annotations. In this work, the optimized structure of berberine was submitted to PharmMapper for prediction of proteins with three dimensional structures in the Protein databank and the target set is just limited to the human targets (2241). The maximum number of reserved matched targets is defined as 300 and all parameters were kept as default.

TCMSP (http://lsp.nwsuaf.edu.cn/tcmsp.php) is a database of systems pharmacology for drug discovery from herbal medicines^59^. The SysDT model was used to predict the potential targets of a compound in TCMSP. SysDT shows impressive performance of prediction for drug-target interactions, with a concordance of 82.83%, a sensitivity of 81.33%, and a specificity of 93.62%, respectively.

### Constructing pathway-target network and drug-targets network

The DAVID^60^ database was used to elucidate the function of potential candidate target proteins related to PCOS in the KEGG biological pathway. To make a deep exploring of the action mechanism of PCOS, the drug-target (D-T) network and pathway-target (P-T) network were constructed by Cytoscape 3.2.2^61^, and the key topological parameter degree and betweeness centrality were analyzed. The degree of a node characters as the number of edges associated with it and the betweeness centrality is equal to the number of shortest paths from all vertices to all others that pass through that node, indicating the importance of the node in a network.

### Molecular docking

To validate the drug-target associations, the molecular docking simulation was further performed on each drug docking with their targets. All drug molecules including berberine were downloaded from Pubchem compound database and transformed to PDB file using Chimera (version 1.10.2). The protein structures of candidate targets were downloaded from RCBS Protein Data Bank (http://www.rcsb.org/pdb) and all protein files were opened with ADT (version 1.5.6) that is the free GUI for AutoDock. Water molecules in each file were deleted and polar hydrogen atoms were added, then wrote to a PDBQT file respectively. We selected the intersection of PCOS potential candidate targets and drugs targets for further research. For validating the interaction and observing the docking sites between drugs and targets, molecular docking was done by an open-source program named AutoDock Vina^62^. The docking results were observed with ADT.

## Additional Information

**How to cite this article**: Wang, Y. *et al*. Systems pharmacology to investigate the interaction of Berberine and other drugs in treating polycystic ovary syndrome. *Sci. Rep.* **6**, 28089; doi: 10.1038/srep28089 (2016).

## Supplementary Material

Supplementary Information

## Figures and Tables

**Table 1:** The clinical drugs for PCOS treatment.

Class	Drug name
COCs	Progesterone
	Estradiol
	Levonorgestrel
	Medroxyprogesterone Acetate
Antiandrogenic progestins	Cyproterone acetate
	Drospirenone
	Dienogest
	Chlormadinone acetate
	Desogestrel
	Gestodene
	Norgestimate
Antiandrogens	flutamide
	Spironolactone
	Finasteride
	Ketoconazole
Insulin-sensitizing drugs	Metformin
	Pioglitazone
	Rosiglitazone
	Liraglutide
	Myo-Inositol
Statins	Atorvastatin
	Rosuvastatin
	Simvastatin
Aromatase inhibitors	letrozole
	anastrozole
Other drugs	Eflornithine
	Clomifene
	Dexamethasone
	Leuprolide
	Triptorelin

**Table 2:** The potential targets of berberine.

PDB ID	Protein name	GENE name	Fit score	Source
1RY0	Aldo-keto reductase family 1 member C3	AKR1C3	3.783	PharmMapper
2AUH	Insulin receptor	INSR	3.771	PharmMapper
1KAV	Tyrosine-protein phosphatase non-receptor type 1	PTPN1	3.759	PharmMapper
1ERE	Estrogen receptor	ESR1	3.756	PharmMapper TCMSP
1GS4	Androgen receptor	AR	3.432	PharmMapper TCMSP
1P93	Glucocorticoid receptor	NR3C1	3.397	PharmMapper
2ZB0	Mitogen-activated protein kinase 14	MAPK14	3.393	PharmMapper
2ACL	Retinoic acid receptor RXR-alpha	RARA	3.389	PharmMapper
2J4A	Thyroid hormone receptor beta	THRB	3.316	PharmMapper
3EY4	Corticosteroid 11-beta-dehydrogenase isozyme 1	HSD11B1	3.261	PharmMapper
1BOA	Methionine aminopeptidase 2	METAP2	3.207	PharmMapper
1E3K	Progesterone receptor	PGR	3.071	PharmMapper
2FJP	Dipeptidyl peptidase 4	DPP4	3.009	PharmMapper
1EXA	Retinoic acid receptor gamma	RARG	2.955	PharmMapper
4GBR	Beta-2 adrenergic receptor	ADRB2		TCMSP

**Table 3:** The results of molecular docking.

Protein name	Drug name	Binding Energy (kcal/mol)
Androgen receptor	Berberine	−8.6
	Chlormadinone acetate	−8.4
	Cyproterone acetate	−8.2
	Dienogest	−7.8
	Drospirenone	−8.7
	Flutamide	−8
	Gestodene	−8.5
	Ketoconazole	−7.4
	4Levonorgestrel	−8.5
	Spironolactone	−7.6
Estrogen receptor	Berberine	−7.3
	Clomifene	−5.7
	Desogestrel	−6.9
	Estradiol	−7.8
	Levonorgestrel	−6.7
	Medroxyprogesterone Acetate	−6.7
	Norgestimate	−7.3
	Progesterone	−7
Progesterone receptor	Berberine	−9.7
	Desogestrel	−7.6
	Drospirenone	−11.9
	Levonorgestrel	−8.7
	Medroxyprogesterone Acetate	−9.5
	Norgestimate	−10
	Progesterone	−10.1
	Spironolactone	−8.6
Glucocorticoid receptor	Berberine	−10
	Dexamethasone	−8.4
	Spironolactone	−8.7
Dipeptidyl peptidase 4	Atorvastatin	−7.9
	Berberine	−8.6
Tyrosine-protein phosphatase non-receptor type 1	Berberine	−7.1
Retinoic acid receptor RXR-alpha	Berberine	−7.7
Retinoic acid receptor gamma	Berberine	−7
Thyroid hormone receptor beta	Berberine	−7.9
Beta-2 adrenergic receptor	Berberine	−7.3
Aldo-keto reductase family 1 member C3	Berberine	−7.6
Corticosteroid 11-beta-dehydrogenase isozyme 1	Berberine	−8.2
Insulin receptor	Berberine	−8.6
Mitogen-activated protein kinase 14	Berberine	−7.7
Methionine aminopeptidase 2	Berberine	−7.2
5′-AMP-activated protein kinase subunit alpha −1	Berberine	−7.3
5′-AMP-activated protein kinase subunit alpha −2	Berberine	−7.0
5′-AMP-activated protein kinase subunit beta −1	Berberine	−8.8
