# Supplementary material for: Systems pharmacology to investigate the interaction of berberine and other drugs in treating polycystic ovary syndrome
Source: Sci Rep. 2016 Jun 16;6:28089. doi: 10.1038/srep28089 (PMC4910093; doi:10.1038/srep28089)
Supplement: Supplementary Information [file srep28089-s1.doc]

Systems pharmacology to investigate the interaction of berberine and other drugs in treating polycystic ovary syndrome

Yu Wang^1^, Xin Fu, Jing Xu^1^, Qiuhong Wang^1^* & Haixue Kuang^1^*

^1^ Key Laboratory of Chinese Materia Medica (Ministry of Education), Heilongjiang University of Chinese Medicine, 150040, Harbin, P.R. China.

* Qiuhong Wang: 2872144234 @qq.com; Haixue Kuang: hxkuang56@163.com

Table S1. Candidate targets of PCOS

| Protein name | GENE name |
| --- | --- |
| Canalicular multispecific organic anion transporter 1 | ABCC2 |
| ATP-binding cassette sub-family C member 8 | ABCC8 |
| Acetyl-CoA carboxylase 2 | ACACB |
| ATP-citrate synthase | ACLY |
| Adenosine receptor A1 | ADORA1 |
| Alpha-1A adrenergic receptor | ADRA1A |
| Alpha-1B adrenergic receptor | ADRA1B |
| Alpha-2A adrenergic receptor | ADRA2A |
| Alpha-2C adrenergic receptor | ADRA2C |
| Beta-1 adrenergic receptor | ADRB1 |
| Beta-2 adrenergic receptor | ADRB2 |
| Beta-3 adrenergic receptor | ADRB3 |
| Agouti-related protein | AGRP |
| Aldo-keto reductase family 1 member C3 | AKR1C3 |
| Androgen receptor | AR |
| Bombesin receptor subtype-3 | BRS3 |
| Carbonic anhydrase 6 | CA6 |
| Cholecystokinin | CCK |
| Cholecystokinin receptor type A | CCKAR |
| Gastrin/cholecystokinin type B receptor | CCKBR |
| CCAAT/enhancer binding protein beta | CEBPB |
| Chloride transport protein 6 | CLCN6 |
| Cannabinoid receptor 1 | CNR1 |
| Ciliary neurotrophic factor receptor subunit alpha | CNTFR |
| Carnitine O-palmitoyltransferase 1, liver isoform | CPT1A |
| Corticotropin-releasing factor binding protein | CRHBP |
| Corticotropin-releasing factor receptor 1 | CRHR1 |
| Corticotropin-releasing factor receptor 2 | CRHR2 |
| Steroid 17-alpha-hydroxylase/17,20 lyase | CYP17A1 |
| Diacylglycerol O-acyltransferase 1 | DGAT1 |
| Dipeptidyl peptidase 4 | DPP4 |
| D(2) dopamine receptor | DRD2 |
| Estrogen receptor | ESR1 |
| Steroid hormone receptor ERR1 | ESRRA |
| Fatty acid-binding protein, epidermal | FABP5 |
| Fatty acid synthase | FASN |
| **Fetuin-B** | FETUB |
| Follicle-stimulating hormone receptor | FSHR |
| Glucose-6-phosphate 1-dehydrogenase | G6PD |
| Gamma-aminobutyric acid receptor subunit alpha-1 | GABRA1 |
| Galanin receptor type 1 | GALR1 |
| Galanin receptor type 2 | GALR2 |
| Galanin receptor type 3 | GALR3 |
| Glucagon receptor | GCGR |
| Glucokinase | GCK |
| Growth hormone receptor | GHR |
| Gastric inhibitory polypeptide | GIP |
| Glucagon-like peptide 1 receptor | GLP1R |
| Glutamate receptor ionotropic, NMDA 1 | GRIN1 |
| Histone deacetylase 4 | HDAC4 |
| Histamine H3 receptor | HRH3 |
| Histamine H4 receptor | HRH4 |
| Corticosteroid 11-beta-dehydrogenase isozyme 1 | HSD11B1 |
| 3 beta-hydroxysteroid dehydrogenase/Delta 5-->4-isomerase type 2 | HSD3B2 |
| 5-hydroxytryptamine receptor 1A | HTR1A |
| 5-hydroxytryptamine receptor 2C | HTR2C |
| 5-hydroxytryptamine receptor 3A | HTR3A |
| Insulin-like growth factor 1 receptor | IGF1R |
| Interleukin-1 beta | IL1B |
| Insulin receptor | INSR |
| Insulin receptor substrate-1 | IRS1 |
| Leptin | LEP |
| Leptin receptor | LEPR |
| Lutropin-choriogonadotropic hormone receptor | LHCGR |
| Lipase | LIP |
| Hormone-sensitive lipase | LIPE |
| Lipase member I | LIPI |
| Lysophosphatidylcholine acyltransferase 1 | LPCAT1 |
| Low-density lipoprotein receptor-related protein 6 | LRP6 |
| Mitogen-activated protein kinase 14 | MAPK14 |
| Mitogen-activated protein kinase 8 | MAPK8 |
| Mitogen-activated protein kinase 9 | MAPK9 |
| Ghrelin O-acyltransferase | MBOAT4 |
| Melanocyte-stimulating hormone receptor | MC1R |
| Melanocortin receptor 3 | MC3R |
| Melanocortin receptor 4 | MC4R |
| Melanin-concentrating hormone receptor 1 | MCHR1 |
| Melanin-concentrating hormone receptor 2 | MCHR2 |
| Methionine aminopeptidase 2 | METAP2 |
| Motilin receptor | MLNR |
| Methylenetetrahydrofolate reductase | MTHFR |
| Arylamine N-acetyltransferase 2 | NAT2 |
| Nicastrin | NCSTN |
| Pro-neuropeptide Y | NPY |
| Neuropeptide Y receptor type 1 | NPY1R |
| Neuropeptide Y receptor type 2 | NPY2R |
| Neuropeptide Y receptor type 4 | NPY4R |
| Neuropeptide Y receptor type 5 | NPY5R |
| Nuclear receptor subfamily 1 group I member 3 | NR1I3 |
| Glucocorticoid receptor | NR3C1 |
| Ornithine decarboxylase 1 | ODC1 |
| Opioid growth factor receptor | OGFR |
| Kappa-type opioid receptor | OPRK1 |
| Mu-type opioid receptor | OPRM1 |
| Progesterone receptor | PGR |
| Cytosolic phospholipase A2 | PLA2G4A |
| Perilipin-1 | PLIN1 |
| Phospholipid transfer protein | PLTP |
| Pancreatic triacylglycerol lipase | PNLIP |
| Peroxisome proliferator activated receptor alpha | PPARA |
| Peroxisome proliferator activated receptor delta | PPARD |
| Peroxisome proliferator activated receptor gamma | PPARG |
| Protein phosphatase 1 regulatory subunit 3A | PPP1R3A |
| 5'-AMP-activated protein kinase catalytic subunit beta-1 | PRKAB1 |
| cAMP-dependent protein kinase type II-beta regulatory subunit | PRKAR2B |
| Presenilin-1 | PSEN1 |
| Presenilin-2 | PSEN2 |
| Gamma-secretase subunit PEN-2 | PSENEN |
| Prostaglandin G/H synthase 1 | PTGS1 |
| Tyrosine-protein phosphatase non-receptor type 1 | PTPN1 |
| Peptide YY | PYY |
| Retinoic acid receptor RXR-alpha | RARA |
| Retinoic acid receptor gamma | RARG |
| RNA-binding protein 28 | RBM28 |
| Sterol regulatory element binding protein cleavage-activating protein | SCAP |
| Corticosteroid-binding globulin | SERPINA6 |
| Solute carrier family 22 member 1 | SLC22A1 |
| Long-chain fatty acid transport protein 1 | SLC27A1 |
| Sodium/glucose cotransporter 2 | SLC5A2 |
| Solute carrier organic anion transporter family member 1B1 | SLCO1B1 |
| 3-oxo-5-alpha-steroid 4-dehydrogenase 1 | SRD5A1 |
| 3-oxo-5-alpha-steroid 4-dehydrogenase 2 | SRD5A2 |
| Thyroid hormone receptor beta | THRB |
| Enteropeptidase | TMPRSS15 |
| Tumor necrosis factor | TNF |
| Tripeptidyl-peptidase 2 | TPP2 |
| Mitochondrial brown fat uncoupling protein 1 | UCP1 |
| Mitochondrial uncoupling protein 2 | UCP2 |
| Mitochondrial uncoupling protein 3 | UCP3 |
| Protein Wnt-4 | WNT4 |

Table S2. Pathway enriching result of candidate targets

| Pathway name | GENE name |
| --- | --- |
| Neuroactive ligand-receptor interaction | ADORA1 |
|  | ADRA1A |
|  | ADRA1B |
|  | ADRA2A |
|  | ADRA2C |
|  | ADRB1 |
|  | ADRB2 |
|  | ADRB3 |
|  | BRS3 |
|  | CCKAR |
|  | CCKBR |
|  | CNR1 |
|  | CRHR1 |
|  | CRHR2 |
|  | DRD2 |
|  | FSHR |
|  | GABRA1 |
|  | GALR1 |
|  | GALR2 |
|  | GALR3 |
|  | GCGR |
|  | GHR |
|  | GLP1R |
|  | GRIN1 |
|  | HRH3 |
|  | HRH4 |
|  | HTR1A |
|  | HTR2C |
|  | LEP |
|  | LEPR |
|  | LHCGR |
|  | MC1R |
|  | MC3R |
|  | MC4R |
|  | MCHR1 |
|  | MCHR2 |
|  | MLNR |
|  | NPY1R |
|  | NPY2R |
|  | NPY5R |
|  | NR3C1 |
|  | OPRK1 |
|  | OPRM1 |
|  | THRB |
| Adipocytokine signaling pathway | ACACB |
|  | AGRP |
|  | CPT1A |
|  | IRS1 |
|  | LEP |
|  | LEPR |
|  | MAPK8 |
|  | MAPK9 |
|  | NPY |
|  | PPARA |
|  | PRKAA1 |
|  | TNF |
| Insulin signaling pathway | ACACB |
|  | FASN |
|  | GCK |
|  | INSR |
|  | IRS1 |
|  | LIP |
|  | MAPK8 |
|  | MAPK9 |
|  | PRKAA1 |
|  | PRKAR2B |
|  | PPP1R3A |
|  | PTPN1 |
| PPAR signaling pathway | CPT1A |
|  | FABP5 |
|  | PLIN1 |
|  | PPARA |
|  | PPARD |
|  | PPARG |
|  | PLTP |
|  | UCP1 |
|  | SLC27A1 |
| Steroid hormone biosynthesis | AKR1C3 |
|  | CYP27B1 |
|  | HSD3B2 |
|  | HSD11B1 |
|  | SRD5A1 |
|  | SRD5A2 |
| Type II diabetes mellitus | ABCC8 |
|  | GCK |
|  | INSR |
|  | IRS1 |
|  | MAPK8 |
|  | MAPK9 |
|  | TNF |
| Calcium signaling pathway | HTR2C |
|  | ADRA1A |
|  | ADRA1B |
|  | ADRB1 |
|  | ADRB2 |
|  | ADRB3 |
|  | CCKAR |
|  | CCKBR |
|  | GRIN1 |
|  | LHCGR |
| Androgen and estrogen metabolism | HSD3B2 |
|  | HSD11B1 |
|  | SRD5A1 |
|  | SRD5A2 |
| Notch signaling pathway | NCSTN |
|  | PSEN1 |
|  | PSEN2 |
|  | PSENEN |
| Progesterone-mediated oocyte maturation | IGF1R |
|  | MAPK14 |
|  | MAPK8 |
|  | MAPK9 |
|  | PGR |
| NOD-like receptor signaling pathway | IL1B |
|  | MAPK14 |
|  | MAPK8 |
|  | MAPK9 |
|  | TNF |
| Fc epsilon RI signaling pathway | MAPK14 |
|  | MAPK8 |
|  | MAPK9 |
|  | PLA2G4A |
|  | TNF |
| Alzheimer's disease | GRIN1 |
|  | IL1B |
|  | PSEN1 |
|  | PSEN2 |
|  | PSENEN |
|  | TNF |
|  | NCSTN |
